# Developmental Predictors of Inattention-Hyperactivity from Pregnancy to Early Childhood

**DOI:** 10.1371/journal.pone.0125996

**Published:** 2015-05-04

**Authors:** Stéphanie Foulon, Jean-Baptiste Pingault, Béatrice Larroque, Maria Melchior, Bruno Falissard, Sylvana M. Côté

**Affiliations:** 1 INSERM U669, University Paris-Descartes and Paris-Sud, Paris, France; 2 Division of Psychology and Language Sciences, University College London, London, United Kingdom; 3 King’s College London, MRC Social, Genetic and Developmental Psychiatry Centre, Institute of Psychiatry, London, United Kingdom; 4 International Laboratory on Child and Adolescent Health Development, University of Montreal, Montreal, Canada; 5 Tomsk state University, Tomsk, Russian Federation; 6 Epidemiology and Clinical Research Unit, Beaujon Hospital, Clichy, France; 7 INSERM UMR S953, Paris, France; 8 INSERM, UMR_S 1136, Pierre Louis Institute of Epidemiology and Public Health, Social Epidemiology Research Team, Paris, France; 9 Sorbonne University, UPMC Univ Paris 06, UMR_S 1136, Pierre Louis Institute of Epidemiology and Public Health, Social Epidemiology Research Team, Paris, France; University of Rennes-1, FRANCE

## Abstract

**Objective:**

The objective of the study was to characterize the developmental sequence of pre- and postnatal risk factors for inattention-hyperactivity symptoms in preschoolers.

**Materials and Methods:**

Longitudinal data came from a French population based birth cohort study (EDEN; N = 1311 mother-child pairs followed from the pregnancy onwards). Inattention-hyperactivity symptoms were assessed with the Strengths and Difficulties Questionnaire when participating children were 3 years of age. Potential risk factors were classified in four domains (fetal exposures and child somatic characteristics, child temperament, child neurodevelopmental status, psychosocial environment) and four periods (before pregnancy, prenatal/birth, infancy, toddlerhood). Their role as potential moderator or mediator was tested with path analysis to determine the developmental sequence.

**Results:**

A low family socioeconomic status before pregnancy was the main environmental risk factor for inattention-hyperactivity symptoms at 3 years, and its effect occurred via two pathways. The first was a risk pathway, where lower SES was associated with higher maternal depression and anxiety during pregnancy; then to higher maternal and child distress and dysregulation in infancy; and in turn to higher levels of inattention-hyperactivity at 3 years. The second was a protective pathway, where higher SES was associated with longer duration of breastfeeding during infancy; then to better child neurodevelopmental status in toddlerhood; and in turn to lower levels of inattention-hyperactivity at 3 years.

**Discussion:**

This study identified psychosocial factors at several developmental periods that represent potential targets for preventing the emergence of inattention-hyperactivity symptoms in early childhood.

## Introduction

High levels of inattention-hyperactivity symptoms during childhood are associated with poor academic and psychosocial outcomes in adulthood [1–4]. Yet to date, little is known about predictors of early symptoms of inattention-hyperactivity, i.e. symptoms appearing prior to age 5 years. Expanding knowledge on the preschool period is crucial for several reasons: 1) recent evidence suggests that a majority of cases of attention deficit hyperactivity disorder have a history of inattention-hyperactivity symptoms prior to school entry [5], 2) early onset is correlated with greater severity of symptoms in school-aged children [6], 3) predictors of inattention-hyperactivity symptoms in preschoolers may not be the same than in school-aged children.

Epidemiologic studies have identified several correlates of inattention-hyperactivity symptoms in preschoolers: parental psychiatric disorders [7–9], low parental education, household poverty, young maternal age at birth, single parenthood [8,9], prenatal maternal exposure to tobacco, alcohol or drugs [8,9], male child sex, very preterm birth [10] and low birth weight [8]. But these studies are scarce and mostly cross-sectional, with frequent retrospective measures of risk factors [7,9]. The few longitudinal studies on this subject have failed to examine the temporal chain of risk factors predicting early inattention-hyperactivity [8,10]. Gathering information on this developmental sequence can help determine on which predictors *and* at which developmental periods we should intervene for a better prevention of the onset of inattention-hyperactivity.

Longitudinal studies typically collect an important amount of data at several time points, raising specific methodological issues. First, the choice of predictors to include in the statistical model at each time point is made difficult by the number of potential (often correlated) risk factors. As such, reducing and classifying risk factors in meaningful categories is necessary. Second, the statistical methods used in most studies do not allow the examination of the temporal chain of risk factors predicting inattention-hyperactivity symptoms. Therefore, there is a need to integrate predictors into a developmental sequence according to their type, e.g. independent predictors, mediators or moderators. For example, a low family socioeconomic status (SES) is associated with inattention-hyperactivity symptoms in preschoolers [8,9]. However, SES is a distal predictor and the pathways from SES to inattention-hyperactivity symptoms are largely unknown. Pre and post-natal factors like maternal depression and mother-child interactions could mediate this contribution of SES to inattention-hyperactivity symptoms. We thus selected a method allowing us to 1) classify the predictors, thus clarifying their distinct roles and 2) identify several potential distal and proximal targets of prevention on the pathway leading to inattention-hyperactivity symptoms.

The objective of the present study was to characterize the developmental sequence of pre and postnatal risk factors for inattention-hyperactivity symptoms in preschoolers using the data from the EDEN study, a population-based birth cohort with prospective data on a wide range of pre and postnatal risk factors.

## Materials and Methods

### Participants

**Fig 1:**
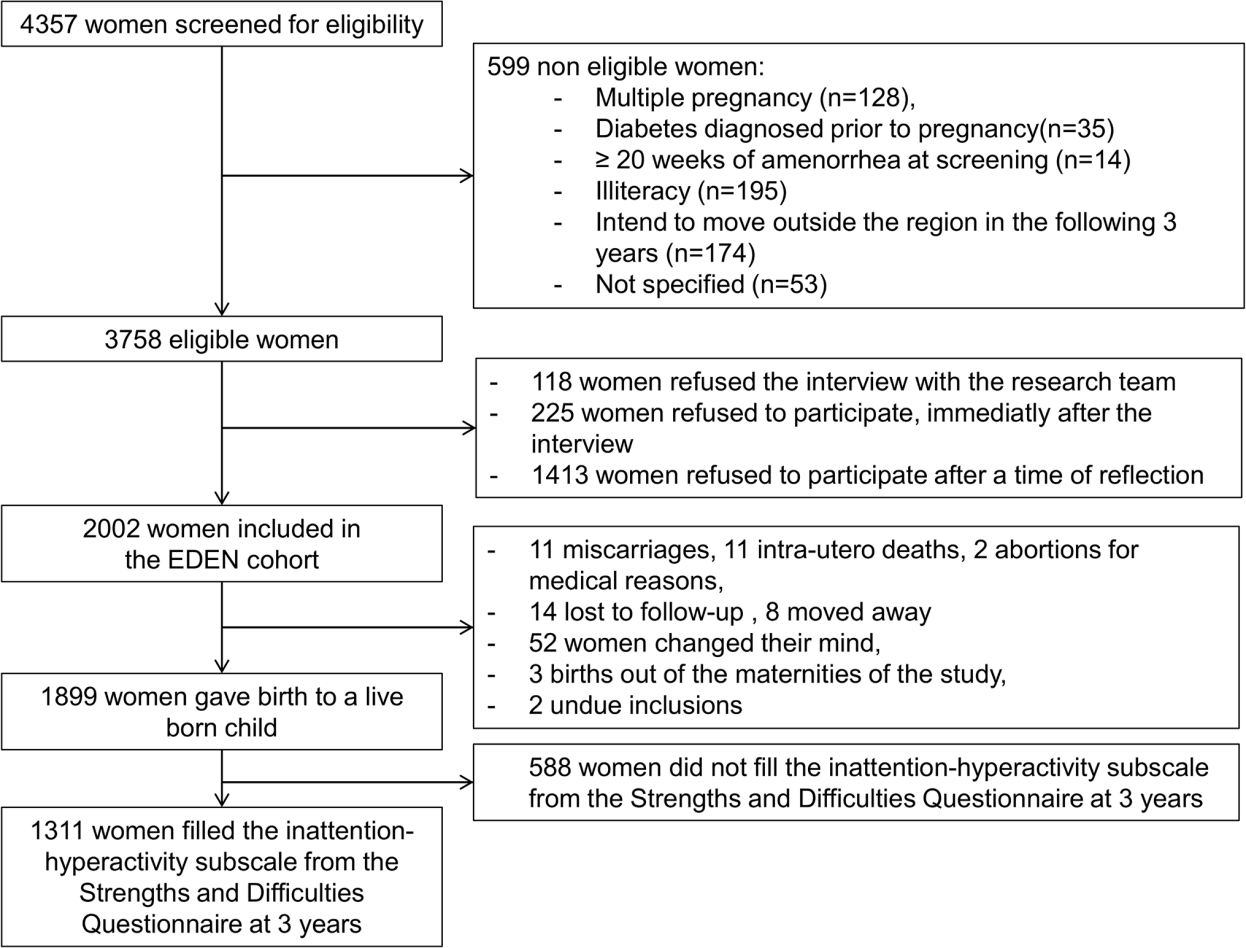
Study population.

Participants of the EDEN mother-child birth cohort study [11] were recruited between 2003–2006 among pregnant women (24 weeks of amenorrhea) followed in two maternity wards in Poitiers and Nancy University hospitals (France). Exclusion criteria were multiple pregnancies (e.g. twins), diabetes diagnosed prior to pregnancy, illiteracy or moving outside the region in the following 3 years. Among women who fulfilled the inclusion criteria, 53% agreed to participate, resulting in the inclusion of 1,899 live born children. The analysis sample includes 1,311 mother-child pairs for whom inattention-hyperactivity subscales from the Strengths and Difficulties Questionnaire were available at 3 years, as shown in Fig 1 [12]. Mothers without inattention-hyperactivity subscale (n = 588) had a significantly lower socioeconomic level, were younger and smoked more than women of live born children with data on inattention-hyperactivity. The study was approved by the Ethics Committee of Kremlin Bicêtre hospital and by the French Data Protection Authority. Written consents were obtained from the mother.

### Measures

All measures were reported by the mother except medical data on pregnancy and delivery, collected by research midwives.

#### Inattention-hyperactivity symptoms at age 3 years

Children’s inattention-hyperactivity symptoms were assessed with the 5-item subscale of the Strengths and Difficulties Questionnaire (SDQ) 3–4, which displays good psychometric properties in preschoolers [12,13]. The items were "Restless, overactive", "Constantly fidgeting", "Easily distracted", "Can stop and think things out before acting" and "Sees tasks through to the end". Items were coded on a 3-points scale from 0 (not true) to 2 (very true) with reverse coding for the two last items. The mean score was 3.5 (SD = 2.2) with a Cronbach coefficient of 0.71.

#### Fetal exposures and child somatic characteristics

Prenatal child exposure to tobacco was assessed by the mean number of cigarettes smoked by the mother during the pregnancy. The mean number of glasses of alcohol per week was estimated separately in the first and third trimester of pregnancy. Prenatal cannabis consumption was collected retrospectively in the post natal care interview, coded from 0 to 4 (every day). Child sex, gestational age at delivery, birth weight, Apgar score at 5 minutes and child resuscitation were extracted from medical records. Duration of breastfeeding was calculated from 4, 8, 12 and 24 months questionnaires. Total child sleep time was assessed at 2 years.

#### Child temperament

The French version of the Infant Characteristics Questionnaire (ICQ) was used to assess children’s temperament at age 4 months [14]. Mothers were asked to rate infant behavior using 23 items scored on a 7-points scale from 1 (more optimal) to 7 (less optimal). Four dimensions of temperament were identified "fussy/difficult", "unpredictable", "inadaptable" and "dull".

#### Child neurodevelopmental status

Neurodevelopmental status was assessed at 2 years by summing items related to gross motor (e.g. “he/she runs”), fine motor (e.g. "he/she knows how to button him/herself") and language (e.g. "he/she makes a 3-word sentence") in the questionnaire [15].

#### Socioeconomic and psychological environment

Family socioeconomic environment was ascertained by monthly household income, maternal age at first child birth and parental educational level (ranging from 1, primary school or less, to 6, postgraduate college). Family structure (i.e. parents living together, number of siblings) was evaluated in pregnancy, infancy and toddlerhood. Childcare at 2 years was coded as non-maternal or maternal. The number of stressful life events occurring in pregnancy and in the first year was obtained by summing events from a list of 17 stressful life events (e.g. death of relative, financial difficulties). Prenatal maternal symptoms of depression and anxiety were assessed during the sixth month of pregnancy using respectively the Center for Epidemiologic Studies Depression Scale (CES-D) and the State Trait Inventory Anxiety (STAI). The CES-D is a 20-item scale on symptoms and behavior characteristic of depressive disorder, rated on a 0–3 scale from "never" to "frequently" [16]. The STAI includes 20 items, scored 0 (lowest degree of anxiety) to 3 (highest) [17]. Postnatal depressive symptoms were evaluated one year after birth using the Edinburgh Postnatal Depression Scale (EPDS) [18].

### Moderator-mediator approach and path analysis

**Fig 2:**
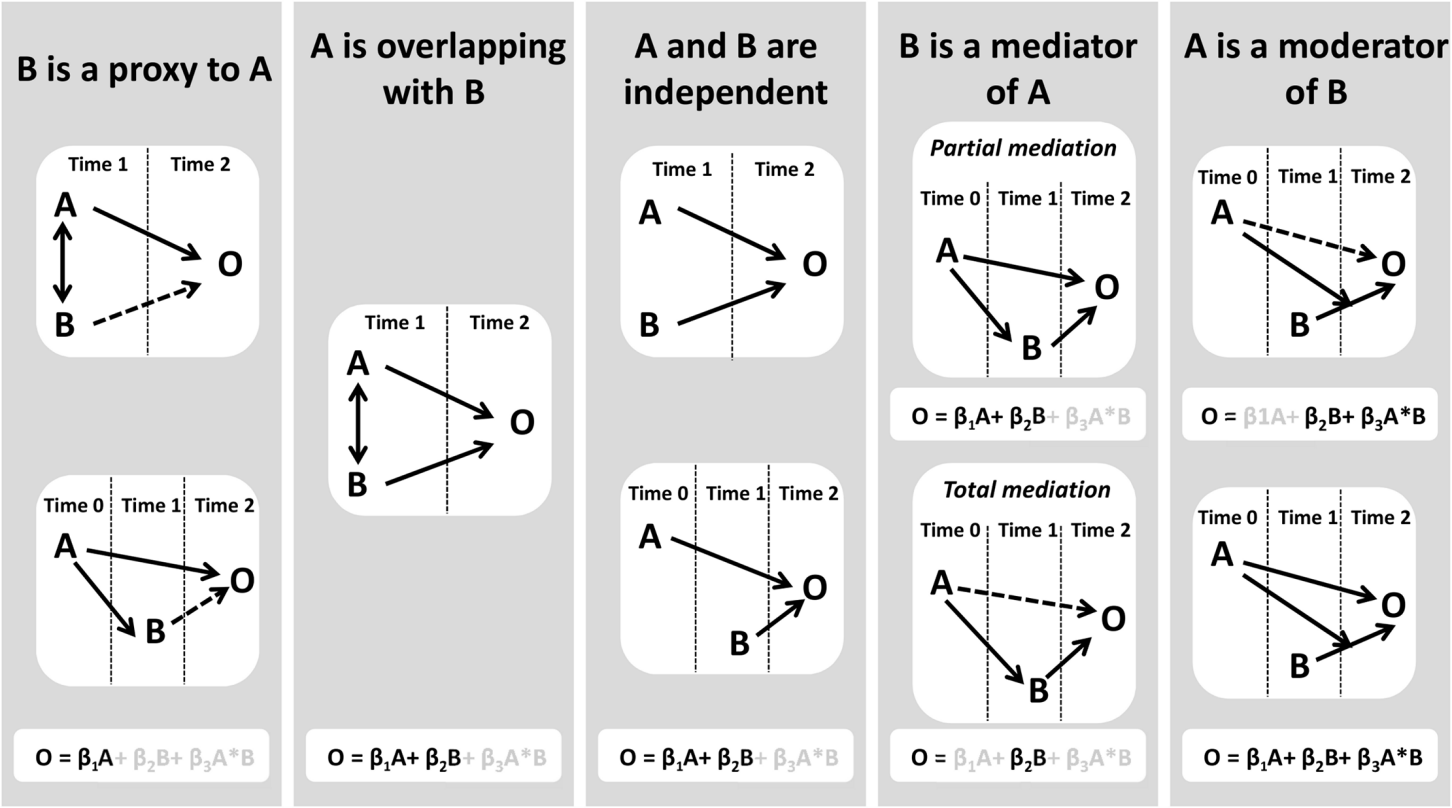
Risk factors classification. A and B are the risk factors. O is the outcome. The single/double-headed arrow between A and B indicates a correlation. The solid (or dotted) arrow between a risk factor and O indicates a significant (or insignificant) association adjusting for the other risk factor.

Cohort studies are a corner stone of epidemiology and provide rich and valuable information. However, using large quantity of information adequately to understand the developmental sequence of risk factors is challenging because many risk factors are correlated, overlapping or reflect spurious associations. In order to address adequately these problems, we analyzed the data using the MacArthur moderator-mediator approach proposed by Kraemer [19]. The approach proposes a classification of risk factors as overlapping, proxy, independent, mediator or moderator risk factors. The classification, illustrated in Fig 2, is based on 1) linear models O = β_1_A+β_2_B+β_3_A*B, where O is the outcome, A and B the risk factors to classify, β_1_, β_2_ and β_3_ the coefficients of regression, 2) temporality between A and B, 3) correlation between A and B.

This method has several advantages. First, it clarifies the distinct role of each risk factor among the mass of information collected during the study. When two risk factors measure different facets of a same construct, combining these "overlapping" risk factors into a single independent risk factor increases the reliability and decreases the number of risk factors included in the analysis. Some of the risk factors have a true association with the outcome ("independent risk factors") whereas others, called "proxy", are spurious. A proxy risk factor is both linked with an independent risk factor and with the outcome but does not predict the outcome by itself. It can confuse the understanding of the real causes of a disorder. Omitting proxy factors can therefore provide a clearer understanding of associations under study. Second, this approach accounts for the temporal order of risk factors and uses all the information available at each time. In models that are usually implemented in epidemiology (e.g. multiple linear regression, logistic regression), all predictors are studied together whatever their temporal relationships. The MacArthur approach takes into consideration such temporal relationships by identifying mediators and moderators. A mediator is an intermediate variable that governs totally or partially the association between a risk factor (temporally preceding the mediator) and the outcome (temporally posterior to the mediator). A moderator is a variable which affects the direction and/or strength of the relation between a risk factor and the outcome.

The use of this structured exploratory approach in a cohort study has been detailed by Essex and follows four steps [20]. In a first step, we organized potential risk factors into four domains (fetal exposures and child somatic characteristics, child temperament, child neurodevelopmental status, psychosocial environment) and four periods (before pregnancy, prenatal/birth, infancy—4, 8 and 12 months—, toddlerhood—24 months). All potential risk factors variables were binary (coded -0.5 and +0.5) or quantitative/ordinal (coded as deviation from the mean). In a second step, we selected the candidate risk factors for inattention-hyperactivity among the potential risk factors of the first step. To be selected, each potential risk factor had to be correlated with the inattention-hyperactivity score at age 3 years (|ρ_spearman_| ≥ 0.10 and p-value<0.05). In a third step, we examined every pair of candidate risk factors, selected in step 2 and belonging to a same period, to classify them as proxy, overlapping or independent risk factors. Proxy variables were omitted from further analyses and a single measure was calculated from the first principal component of the overlapping variables. Analyses were conducted within domains and then across domains, to obtain a set of independent risk factors within each time period. In the fourth step, after omitting proxy factors between periods, we connected risk factors across time and identified them as independent, mediator or moderator risk factors. The presence of a moderator implies to stratify the sample and to start again the analysis from the 2^nd^ step on the subgroups defined by the moderator. These four steps result in one or several developmental sequences (depending on the presence of moderators) with pathways linking the risk factors over time to inattention-hyperactivity symptoms at 3 years. To assess the relationships between the identified risk factors in a multivariate model, we performed a path analysis [21]. The direction and the strength of relationships between study variables were estimated using standardized path coefficients. The fit between the model and the data was assessed with the chi square test of the model, the Root Mean Square Error of Approximation (RMSEA), the Comparative Fit Index (CFI), and the Standardized Root Mean Square Residual (SRMR). RMSEA < 0.05, SRMR < 0.08 and CFI > 0.95 were considered to indicate a good fit [22,23].

## Results

### Organisation into domains and periods (1st step) and selection of risk factors (2nd step)

Table 1 presents the 43 potential risk factors explored, split into domains and periods. Descriptive statistics of these variables are shown in S1 Table. Among the potential risk factors, 13 met the pre specified statistical criteria (|ρ_spearman_| ≥ 0.10 and p-value<0.05) and were retained for further analyses.

**Table 1:** Potential risk factors for inattention-hyperactivity symptoms at 3 years.

DOMAINS	BEFORE PREGNANCY	PREGNANCY BIRTH	INFANCY (4-8-12 MONTHS)	TOODLERHOOD (24 MONTHS)
**Fetal exposures and child somatic characteristics**		**Mean number of cigarettes smoked during pregnancy (+0.13)**	**Breastfeeding duration (-0.13)**	Child total sleep time (-0.06)
		**Child sex (+0.11)**		
		Mean number of alcohol glasses/week at first trimester (+0.06)		
		Child birth weight (-0.06)		
		Mean number of alcohol glasses/week at third trimester (+0.04)		
		Child gestational age at birth (-0.03)		
		Cannabis consumption during pregnancy (0.00)		
		Apgar score at 5 min (0.00)		
		Child resuscitation (0.00)		
**Child temperament**			**Fussy/Difficult (+0.12)** ^**c**^	
			Unpredictable (+0.08)	
			Inadaptable (+0.06)	
			Dull (-0.03)	
**Child neurodevelopment**				**Fine motor (-0.17)** ^**d**^
				**Language (-0.14)** ^**d**^
				Gross motor (+0.03)
**Family psychosocial environment**	**Maternal educational level (-0.24)** ^**a**^	**Maternal depression symptoms (+0.14)** ^**b**^	**Post-partum depression symptoms (+0.12)** ^**c**^	Maternal child care (+0.06)
	**Paternal educational level (-0.22)** ^**a**^	**Maternal anxiety symptoms (+0.12)** ^**b**^	Number of stressful life events (+0.09)	Parents living together (-0.05)
	**Monthly family income (-0.18)** ^**a**^	**Maternal age at birth (-0.12)**	Number of children with whom the child is cared (+0.08)	Number of psychiatrist or psychologist consultations (-0.03)
	Maternal age at first child (-0.09)	Number of stressful life events (+0.07)	Number of psychiatrist or psychologist consultations (+0.07)	Maternal psychoactive drugs intake (+0.01)
	Maternal history of hospitalization in psychiatry (+0.04)	Parents living together (-0.06)	Maternal psychoactive drugs intake (+0.07)	
	Psychiatrist or psychologist consultation in the year before pregnancy (0.00)	Psychiatric histories in obstetrical record (+0.02)	Number of siblings (-0.05)	
		Maternal psychoactive drugs intake (+0.02)	Parents living together (-0.04)	
			Paternal involvement (-0.03)	

Potential risk factors are presented in their domain and period (1^st^ step). Selected risk factors (in bold) are correlated with inattention-hyperactivity score at 3 years with |ρ|≥0.10 (2^nd^ step). Some risk factors are overlapping and combined in

^a^"Socio Economic Status"

^b^"Maternal anxiety depression symptoms"

^c^"Maternal and Child distress and dysregulation" and

^d^"Neurodevelopmental status" (3^rd^ step).

### Classification of risk factors into proxy, overlapping or independent variables within each period (3rd step)

Monthly family income and paternal and maternal education were overlapping factors. They were therefore combined into a single measure referred to as "Socio-Economic Status "(SES). Similarly, prenatal maternal symptoms of depression and anxiety were overlapping factors in the prenatal/birth period. They were therefore combined into a "Maternal Anxiety and Depression" measure. Because the mean number of cigarettes smoked by the mother during pregnancy and maternal age at birth were only somewhat overlapping (ρ_spearman_ = -0.17) and there was no conceptual justification for the agglomerating of these variables into a single measure, we treated them as independent risk factors. Child male sex was an independent risk factor of inattention-hyperactivity symptoms. In the infancy period, maternal depressive symptoms and the child’s difficult temperament were overlapping and combined into a "Mother and Child distress and dysregulation" composite variable. Infant breastfeeding duration was an independent protective factor of inattention-hyperactivity symptoms. In the toddlerhood period (2 years), fine motor score and language score were overlapping and therefore combined into a "Neurodevelopmental status" measure.

### Developmental sequence (4th step)

**Fig 3:**
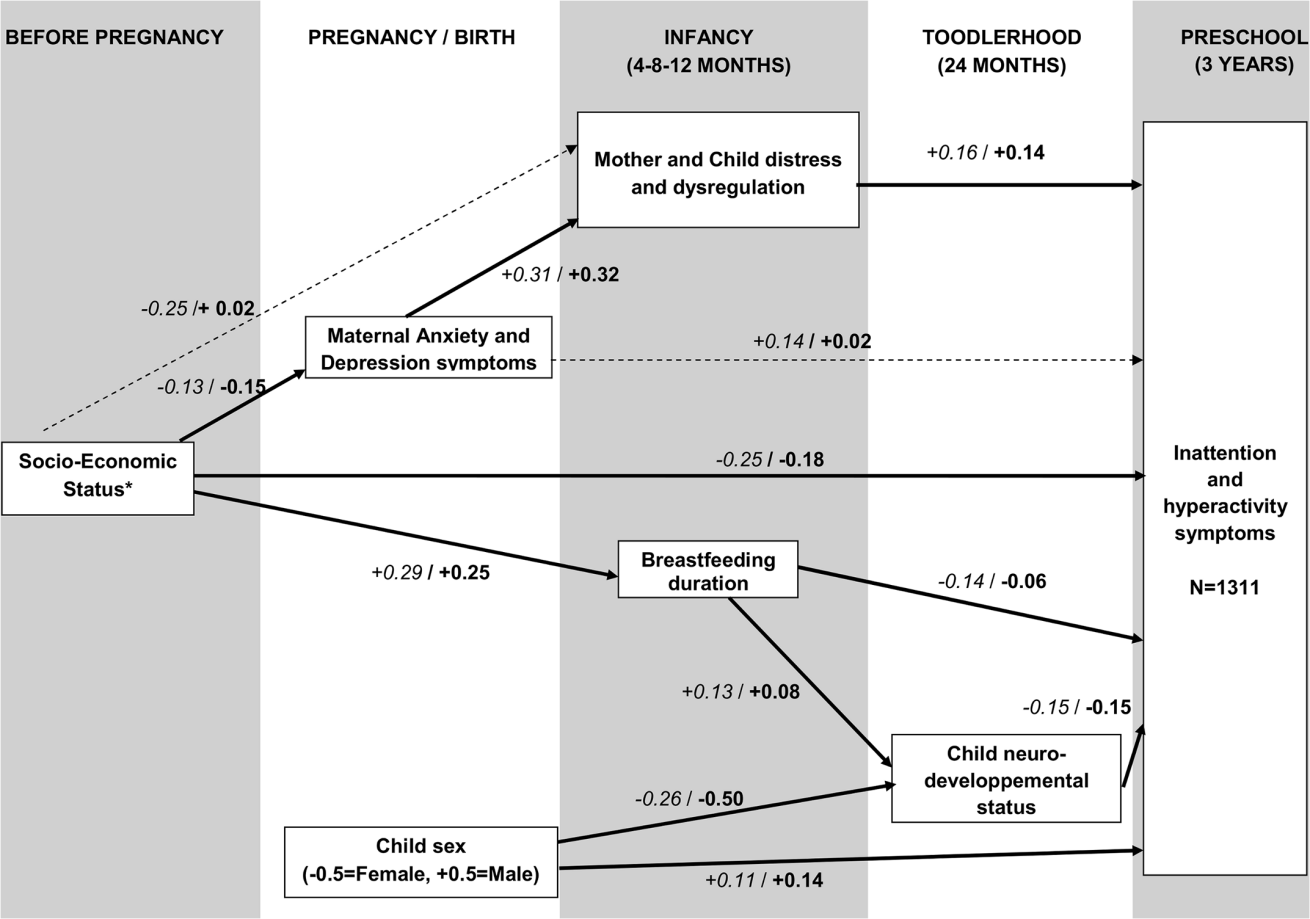
Longitudinal model of inattention-hyperactivity symptoms emergence (4^th^ step). *Prenatal tobacco and maternal age at birth were proxies of SES. Italicized coefficients are bivariate correlation coefficients. Bold coefficients are standardized path coefficients. A solid arrow indicates a statistical significance (p<0.05) of the multivariate path coefficient A dotted arrow indicates a bivariate significance that disappears in the multivariate model.

When connecting risk factors across time, we identified the mean number of cigarettes smoked during pregnancy and maternal age at birth as proxies of SES and therefore omitted them from further analyses. There was no moderator among the risk factors studied. The developmental sequence with bivariate correlation coefficients and standardized path coefficients between risk factors and the inattention-hyperactivity score is shown in Fig 3. Children from low SES family were more likely to have inattention-hyperactivity symptoms. The effect of this factor was partially mediated by mother’s anxiety and depression during pregnancy, which predicted maternal and child distress and dysregulation in infancy. Male sex was an independent risk factor of inattention-hyperactivity symptoms. To the opposite, a longer breastfeeding duration and a better neurodevelopmental status were associated with fewer inattention-hyperactivity symptoms. The effect of child’s sex and breastfeeding duration were partially mediated by the neurodevelopmental status. The model fitted the data adequately (χ2 = 14.1, df = 8, p-value = 0.08, CFI = 0.98, RMSEA = 0.02, SRMR = 0.024).

## Discussion

The purpose of this study was to map the developmental sequence of pre and early post natal predictors for inattention-hyperactivity symptoms in early childhood in a population- based birth cohort followed from the second semester of pregnancy to age 3 years. A low socioeconomic status was the main environmental risk factor of inattention-hyperactivity symptoms at age 3 years and its effect occurred via two pathways. The first was a risk pathway: lower SES was associated with higher maternal depression and anxiety during pregnancy; then to higher maternal and child distress and dysregulation in infancy; and in turn to higher levels of inattention-hyperactivity at 3 years. The second was a protective pathway: higher SES was associated with longer duration of breastfeeding during infancy; then to better child neurodevelopmental status in toddlerhood; and in turn to lower levels of inattention- hyperactivity at 3 years.

Contrary to Essex et al., we did not identify SES to be a moderator [20]. Differences in the characteristics of their study, namely in the outcome (a score of severity of mental health symptoms), in the level of the statistical criteria (|ρ_spearman_| ≥ 0.20) and in the type of informant for the measures (teachers and direct observation) may explain this discrepancy. But the current study provides additional evidence for the association between low socioeconomic status and higher levels of inattention-hyperactivity symptoms. These findings support the results of several studies [24–28] conducted in school-aged children and stress the importance of adverse family environment as a source of risk of inattention-hyperactivity symptoms. However, this study gives new insights regarding the nature of the SES/inattention-hyperactivity association by identifying two pathways for the effect of SES.

First, the effect of SES occurred via maternal perinatal depression and impaired mother-child relationships. Interestingly, the contribution of maternal anxiety and depression symptoms during pregnancy to inattention-hyperactivity was fully mediated by mother and child distress. A classical approach would have probably concluded that anxiety and depression symptoms during pregnancy were not independent predictors of inattention-hyperactivity, thereby disqualifying them as potential intervention targets. This highlights the importance of using adequate methods to identify distal and proximal potential targets. Moreover, the timing of prevention strategies is an important consideration in the implementation of prevention programs, as illustrated by Donovan and Spence in children with anxiety disorders [29]. Drawing the map of the developmental sequence of pre and early post natal risk factors may help to work out more effective preventive strategies.

Second, the association between SES and inattention-hyperactivity was mediated by the duration of breastfeeding. The causal nature of the association between breastfeeding and behaviors is debated. While many studies have reported that breastfed children display less behavioral problems, even after statistically adjusting for demographic factors [30], the promotion of breastfeeding through intervention programs has not yielded positive effects on inattention-hyperactivity [31]. Additional research is needed to establish whether the promotion of breastfeeding may be beneficial in some groups of children (for instance those born to low-SES families, or those in which the mother experiences depression).

The choice of a dimensional measure (score of inattention-hyperactivity symptoms) rather than a categorical one (diagnosis of ADHD yes/no) may be discussed. ADHD is rarely diagnosed as early as 3 years of age because behaviors of inattention-hyperactivity leading to the diagnosis may be transient or reflect normative temperamental variations at this age. However, children with early inattention-hyperactivity symptoms who do not meet all the criteria for ADHD diagnosis are still at risk of academic underachievement in elementary school [3]. Therefore, predictors of inattention-hyperactivity symptoms in preschoolers deserve to be studied. Furthermore, there is considerable evidence that ADHD is a dimensional trait rather than a categorical disorder [32] and the dimensional approach affords a number of benefits, including the preservation of more information, superior reliability, and greater power in statistical analyses.

Limitations should be considered when interpreting the present results. First, our sample is socially more privileged than the French general population. As a result, the range of socioeconomic circumstances in our study is not as wide as in the general population, and the association between socioeconomic status and children’s behavior may be stronger than we report. Second, we relied on maternal reports of children’s behavior, which may be influenced by maternal psychopathology. In future investigations paternal or caregiver reports of children’s hyperactivity and inattention would be useful. Third, the design of the study was not genetically informative so that we could not control for potential passive gene-environment correlations explaining the relationship between SES and inattention-hyperactivity symptoms [33]. Fourth, we cannot conclude on the causal nature of the risk factors thus identified, on the inattention-hyperactivity symptoms at 3 years. Our approach is exploratory, useful to generate hypotheses. These hypotheses have to be confirmed by other epidemiological studies and randomized controlled trials proposing intervention to counteract the risk factors identified and reinforce the protective factors.

Despite these limitations, our study has a number of strengths including the use of a population sample with a longitudinal prospective design, a large prenatal collection of maternal exposures, validated psychosocial assessments and a methodology providing a developmental sequence, thus clarifying potential underlying mechanisms and identifying potential targets.

In summary, children who grow up in low SES families have higher levels of inattention-hyperactivity symptoms as early as in preschool. We identified two pathways by which the effect of SES occurred. If these results are replicated, the next step will be to evaluate in experimental studies the effect of intervention on socioeconomic adversity, improved screening and treatment of maternal perinatal depression and promotion of breastfeeding.

## Supporting Information

S1 Table Descriptive statistics of the potential risk factors of inattention-hyperactivity symptoms at 3 years. ρ is the spearman correlation coefficient between the variable and the score of inattention-hyperactivity symptoms at 3 years. MD: Missing Data, SD: Standard Deviation, T1: first trimester, T3: third trimester, CES-D: Center for Epidemiologic Studies Depression, STAI: State Trait Inventory Anxiety, ICQ: Infant Characteristics Questionnaire, EPDS: Edinburgh Postnatal Depression Scale.
(DOC)
