# Supplementary material for: Developmental Predictors of Inattention-Hyperactivity from Pregnancy to Early Childhood
Source: PLoS One. 2015 May 4;10(5):e0125996. doi: 10.1371/journal.pone.0125996 (PMC4418828; doi:10.1371/journal.pone.0125996)
Supplement: S1 Table — ρ is the spearman correlation coefficient between the variable and the score of inattention-hyperactivity symptoms at 3 years. MD: Missing Data, SD: Standard Deviation, T1: first trimester, T3: third trimester, CES-D: Center for Epidemiologic Studies Depression, STAI: State Trait Inventory Anxiety, ICQ: Infant Characteristics Questionnaire, EPDS: Edinburgh Postnatal Depression Scale. (DOC) [file pone.0125996.s001.doc]

S1 Table. Descriptive statistics of the potential risk factors of inattention-hyperactivity symptoms at 3 years.

ρ is the spearman correlation coefficient between the variable and the score of inattention-hyperactivity symptoms at 3 years. MD: Missing Data, SD: Standard Deviation, T1: first trimester, T3: third trimester, CES-D: Center for Epidemiologic Studies Depression, STAI: State Trait Inventory Anxiety, ICQ: Infant Characteristics Questionnaire, EPDS: Edinburgh Postnatal Depression Scale.

| Domain | Variable | N (%) | MD | Mean (SD) | Ρ |
| --- | --- | --- | --- | --- | --- |
| Before pregnancy | | | | | |
| Psychosocial environnement | Maternal educational level |  | 19 |  | -0.24 |
|  | Primary school | 58 (5) |  |  |  |
|  | Middle school | 233 (18) |  |  |  |
|  | Technical high school | 145 (11) |  |  |  |
|  | General high school | 89 (7) |  |  |  |
|  | Undergraduate college | 300 (23) |  |  |  |
|  | Postgraduate college | 467 (36) |  |  |  |
|  | Paternal educational level |  | 44 |  | -0.22 |
|  | Primary school | 88 (7) |  |  |  |
|  | Middle school | 296 (23) |  |  |  |
|  | Technical high school | 151 (12) |  |  |  |
|  | General high school | 87 (7) |  |  |  |
|  | Undergraduate college | 287 (23) |  |  |  |
|  | Postgraduate college | 358 (28) |  |  |  |
|  | Monthly family income |  | 6 |  | -0.18 |
|  | ≤ 800 € | 36 (3) |  |  |  |
|  | 801-1500 € | 114 (9) |  |  |  |
|  | 1501-2300 € | 388 (30) |  |  |  |
|  | 2301-3000 € | 376 (29) |  |  |  |
|  | 3001-3800 € | 226 (17) |  |  |  |
|  | 3801-4500 € | 108 (8) |  |  |  |
|  | > 4500 € | 54 (4) |  |  |  |
|  | Maternal age at first child | 1311 | 0 | 28.9 (4.6) | -0.09 |
|  | Maternal history of hospitalization in psychiatry |  | 5 |  | +0.04 |
|  | Yes | 34 (3) |  |  |  |
|  | No | 1272 (97) |  |  |  |
|  | Psychiatrist or psychologist consultation in the year before pregnancy |  | 7 |  | 0.00 |
|  | Yes | 127 (10) |  |  |  |
|  | No | 1177 (90) |  |  |  |
| Pregnancy Birth period | | | | | |
| Fetal exposures and child somatic characteristics | Mean number of cigarettes smoked during pregnancy | 1279 | 32 | 1.1 (2.8) | +0.13 |
|  | Child sex |  | 0 |  | +0.11 |
|  | Male | 690 (53) |  |  |  |
|  | Female | 621 (47) |  |  |  |
|  | Mean number of alcohol glasses/week at T1 | 826 | 485 | 0.4 (1.7) | +0.06 |
|  | Child birth weight | 1311 | 0 | 3293 (495) | -0.06 |
|  | Mean number of alcohol glasses/week at T3 | 1245 | 66 | 1.1 (3.3) | +0.04 |
|  | Child gestational age at birth | 1311 | 0 | 39.3 (1.7) | -0.03 |
|  | Cannabis consumption during pregnancy : |  | 33 |  | 0.00 |
|  | No consumption | 1262 (99) |  |  |  |
|  | Less than once a month | 8 (1) |  |  |  |
|  | 1-4 times a month | 4 (0) |  |  |  |
|  | 1-4 times a week | 4 (0) |  |  |  |
|  | Apgar score at 5 mn | 1286 | 25 | 9.7 (0.7) | 0.00 |
|  | Child resuscitation |  | 9 |  | 0.00 |
|  | Yes | 110 (8) |  |  |  |
|  | No | 1192 (92) |  |  |  |
| Psychosocial environnement | Maternal depression symptoms (CES-D) | 1301 | 10 | 11 (7.5) | +0.14 |
|  | Maternal anxiety symptoms (STAI) | 1307 | 4 | 10.1 (9.5) | +0.12 |
|  | Maternal age at birth | 1311 | 0 | 30.4 (4.7) | -0.12 |
|  | Number of stressful life events | 1289 | 22 | 0.2 (0.5) | +0.07 |
|  | Parents living together |  | 8 |  | -0.06 |
|  | No | 53 (4) |  |  |  |
|  | Yes | 1250 (96) |  |  |  |
|  | Psychiatric histories in obstetrical record |  | 1 |  | +0.02 |
|  | Yes | 34 (3) |  |  |  |
|  | No | 1276 (97) |  |  |  |
|  | Maternal psychoactive drugs intake |  | 0 |  | +0.02 |
|  | Yes | 96 (7) |  |  |  |
|  | No | 1215 (93) |  |  |  |
| Infancy period (4-8-12 months) | | | | | |
| Fetal exposures and child somatic characteristics | Breastfeeding duration | 1215 | 96 | 95 (116) | -0.13 |
| Child temperament | Fussy/Difficult ICQ subscale | 1272 | 39 | 13.6 (6.1) | +0.12 |
|  | Inadaptable ICQ subscale | 1270 | 41 | 4.9 (4.0) | +0.06 |
|  | Unpredictable ICQ subscale | 1275 | 36 | 6.6 (3.7) | +0.08 |
|  | Dull ICQ subscale | 1274 | 37 | 2.6 (2.3) | -0.03 |
| Psychosocial environnement | Post-partum depression symptoms (EPDS) | 1231 | 80 | 4.5 (4.6) | +0.12 |
|  | Number of stressful life events | 1229 | 82 | 1 (1.1) | +0.09 |
|  | Number of children with whom the child is cared | 557 | 754 | 2.2 (1.3) | +0.08 |
|  | Number of psychiatrist or psychologist consultations | 1162 | 149 | 0.5 (2.7) | +0.07 |
|  | Maternal psychoactive drug intake |  | 13 |  | +0.07 |
|  | Yes | 118 (9) |  |  |  |
|  | No | 1180 (91) |  |  |  |
|  | Number of siblings | 1185 | 126 | 0.8 (1.0) | -0.05 |
|  | Parents living together |  | 4 |  | -0.04 |
|  | Yes | 1244 (95) |  |  |  |
|  | No | 63 (5) |  |  |  |
|  | Paternal involvement | 1264 | 47 | 2.1 (0.6) | -0.03 |
| Toddlerhood (24 months) | | | | | |
| Fetal exposures and child somatic characteristics | Child total sleep time | 1090 | 221 | 13.1 (1.0) | -0.06 |
| Child neurodevelopment | Fine motor score | 1129 | 182 | 3.7 (1.0) | -0.17 |
|  | Language score | 1161 | 150 | 2.8 (1.5) | -0.14 |
|  | Gross motor score | 1173 | 138 | 4.7 (0.6) | +0.03 |
| Psychosocial environnement | Maternal child care |  |  |  | +0.06 |
|  | Yes | 121 (12) |  |  |  |
|  | No | 870 (88) |  |  |  |
|  | Parents living together |  | 119 |  | -0.05 |
|  | Yes | 1132 (95) |  |  |  |
|  | No | 60 (5) |  |  |  |
|  | Number of psychiatrist or psychologist consultations | 1172 | 139 | 0.5 (2.9) | -0.03 |
|  | Maternal psychoactive drugs intake |  | 145 |  | +0.01 |
|  | Yes | 85 (7) |  |  |  |
|  | No | 1081 (93) |  |  |  |
